# Pollinator-Mediated Selection on Flower Color, Flower Scent and Flower Morphology of *Hemerocallis*: Evidence from Genotyping Individual Pollen Grains On the Stigma

**DOI:** 10.1371/journal.pone.0085601

**Published:** 2013-12-23

**Authors:** Shun K. Hirota, Kozue Nitta, Yoshihisa Suyama, Nobumitsu Kawakubo, Akiko A. Yasumoto, Tetsukazu Yahara

**Affiliations:** 1 Department of Biology, Faculty of Sciences, Kyushu University, Fukuoka, Japan; 2 Field Science Center, Graduate School of Agricultural Science, Tohoku University, Osaki, Miyagi, Japan; 3 Department of Environmental Science, Faculty of Applied Biological Sciences, Gifu University, Gifu, Japan; Lund University, Sweden

## Abstract

To trace the fate of individual pollen grains through pollination processes, we determined genotypes of single pollen grains deposited on *Hemerocallis* stigmas in an experimental mixed-species array. *Hemerocallis fulva*, pollinated by butterflies, has diurnal, reddish and unscented flowers, and *H. citrina*, pollinated by hawkmoths, has nocturnal, yellowish and sweet scent flowers. We observed pollinator visits to an experimental array of 24 *H. fulva* and 12 F2 hybrids between the two species (*H. fulva* and *H. citrina*) and collected stigmas after every trip bout of swallowtail butterflies or hawkmoths. We then measured selection by swallowtail butterflies or hawkmoths through male and female components of pollination success as determined by single pollen genotyping. As expected, swallowtail butterflies imposed selection on reddish color and weak scent: the number of outcross pollen grains acquired is a quadratic function of flower color with the maximum at reddish color, and the combined pollination success was maximal at weak scent (almost unrecognizable for human). This explains why *H. fulva*, with reddish flowers and no recognizable scent, is mainly pollinated by swallowtail butterflies. However, we found no evidence of hawkmoths-mediated selection on flower color or scent. Our findings do not support a hypothesis that yellow flower color and strong scent intensity, the distinctive floral characteristics of *H. citrina*, having evolved in adaptations to hawkmoths. We suggest that the key trait that triggers the evolution of nocturnal flowers is flowering time rather than flower color and scent.

## Introduction

Plants exhibit a tremendous diversity of floral traits that are often highly differentiated among closely related species. Among these floral traits, flower color [1-3] and floral scent [4-6] function to attract particular pollinators, and floral morphology, such as corolla shape and anther-stigma distance, determines the efficiency of pollen transfer [7-9]. Thus, it is widely believed that flower color, floral scent and floral morphology have evolved under pollinator-mediated selection on those traits [10,11]. However, measurements of pollinator-mediated selection on those traits are still limited [12], reflecting the difficulty of tracing the fates of individual pollen grains through pollination processes [13,14]. 

To quantify pollinator-mediated selection on floral traits, we need to measure both female and male fitness components, the number of seeds produced and the number of seeds sired because the majority of flowers are hermaphrodite and have both male and female functions. For male fitness components, many studies have used surrogate measures of siring success [15-19], sometimes leading to biased estimates of male fitness [16]. These surrogates include insect visitation rates [17], pollen removal [18], and pollen dye transfer [19]. In several recent studies, male fitness was measured more directly by paternity analysis of seeds [20-22]. Although the number of seeds sired estimated by paternity analysis is considered as a more reliable estimate of male fitness, it is affected not only by pollination process, but also by fertilization processes where available resources and pollen-pistil interaction are required for the fertilization success. To measure pollinator-mediated selection on floral traits, therefore, it is desirable to estimate paternal success by excluding the influences of resource limitation and pollen-pistil interaction. To obtain such estimates, we employ microsatellite genotypes of single pollen grains [23] that allows us to determine the donor of each pollen grain deposited on stigmas. 

Hybrid population approaches have been provided extraordinary opportunities for measuring pollinator-mediated selection on floral traits as segregation of genes for different floral traits is determined in sister species [1,24,25]. Bradshaw and Schemske [1] demonstrated that a single allele substitution at a flower color gene of two closely related *Mimulus* species, *M. lewisii*, a bumblebee-pollinated species, and *M. cardinalis*, a hummingbird-pollinated species, results in attracting hummingbirds and can initiate pollinator shift from bumblebees to hummingbirds. For floral scent, Klahre et al. [25] used introgression lines between *Petunia exserta* and *P. axillaris*, having a typical hummingbird and hawkmoth syndrome, respectively and demonstrated that new emission of floral scent caused by substitutions of two major genetic loci was effective to attract nocturnal hawkmoths. This finding suggests that mutations in floral scent can cause shifts from diurnal pollination to nocturnal pollination. While these pioneering studies demonstrating pollinator-mediated selection on flower color and scent using hybrid populations, however, they did not examine pollinator-mediated selection on floral morphology despite the fact that sister species of *Mimulus* [1] and *Petunia* [25] markedly differ in floral traits. A pollinator shift may drive the evolution of floral traits as mechanical fit to a new pollinator through intermediate "stepping stones" towards some adaptive optima [7]. Hybrid populations in which genes determining "mechanical fits" to different pollinators are segregated would again provide extraordinary opportunities for testing how pollinators impose selection on phenotypes associated with "mechanical fit".

In this paper, we evaluate the selection mediated by diurnal and nocturnal pollinators on attraction and morphological traits in a hybrid population between Daylily (*Hemerocallis fulva*) and Nightlily (*H. citrina*). *H. fulva* is swallowtail butterflies-pollinated species with diurnal, reddish, unscented flowers and *H. citrina* is crepuscular and nocturnal hawkmoths-pollinated species with yellowish flower and sweet scent [26-28]. Flower longevity of two species is approximately half a day. Flowering of two species overlaps in the evening from 16:30 to 21:30 [26,27]. During this time zone of flowering overlap, both swallowtail butterflies and hawkmoths actively forage on flower nectar of two species and their hybrids [28]. Thus, selection imposed by both swallowtail butterflies and hawkmoths would have played a significant role of driving the evolution from a *H. fulva*-like diurnally flowering ancestor to a nocturnal flowering species, *H. citrina* [26,27]. To deepen our understanding of this evolutionary process, F2 hybrids of two species have been generated [29]. Both F1 and F2 hybrids of two species are highly fertile and floral traits, including color, scent and position of reproductive organs, are segregated. Utilizing the opportunity provided by these F2 hybrids, we recorded visits of swallowtail butterflies and hawkmoths to an experimental array mixed with a parental species and F2 hybrids and then estimated outcross pollen acquisition and donation of individual plants by determining genotypes of individual pollen grains deposited on stigma. In this way, we directly measured selection by butterflies and hawkmoths through both pollen receipt (i.e., maternal success) and pollen donation success (i.e., paternal success). The purpose of this paper is to report our findings from this measurement. Specifically, we aimed at evaluating how the attractive floral traits (tepal color and floral scent) and morphological traits (floral morphology) are selected by swallowtail butterflies and hawkmoths.

## Materials and Methods

### Plants used in the experiments

Plants of the butterfly pollinated *Hemerocallis fulva* L. var. *aurantiaca* (Baker) M. Hotta, were collected in Haifuku (Hirado island, Nagasaki Prefecture, Japan). *H. citrina* var. *vespertina* (H. Hara) M. Hotta was collected in Tsutsumi about 10km NE of Haifuku (for details, see 30). No specific permissions were required as the locations are not protected in any way nor did our collections involve endangered or protected species. To produce F1 hybrids, *H. fulva* plants were hand-pollinated by pollen of *H. citrina* in 2001 [29]. To produce F2 hybrids, F1 plants were hand-pollinated by pollen of full sibling F1 plants in 2003 and 2004 [27]. All plants were grown in pots in the nursery of Department of Biology, Kyushu University (Fukuoka, Japan).

**Figure 1:**
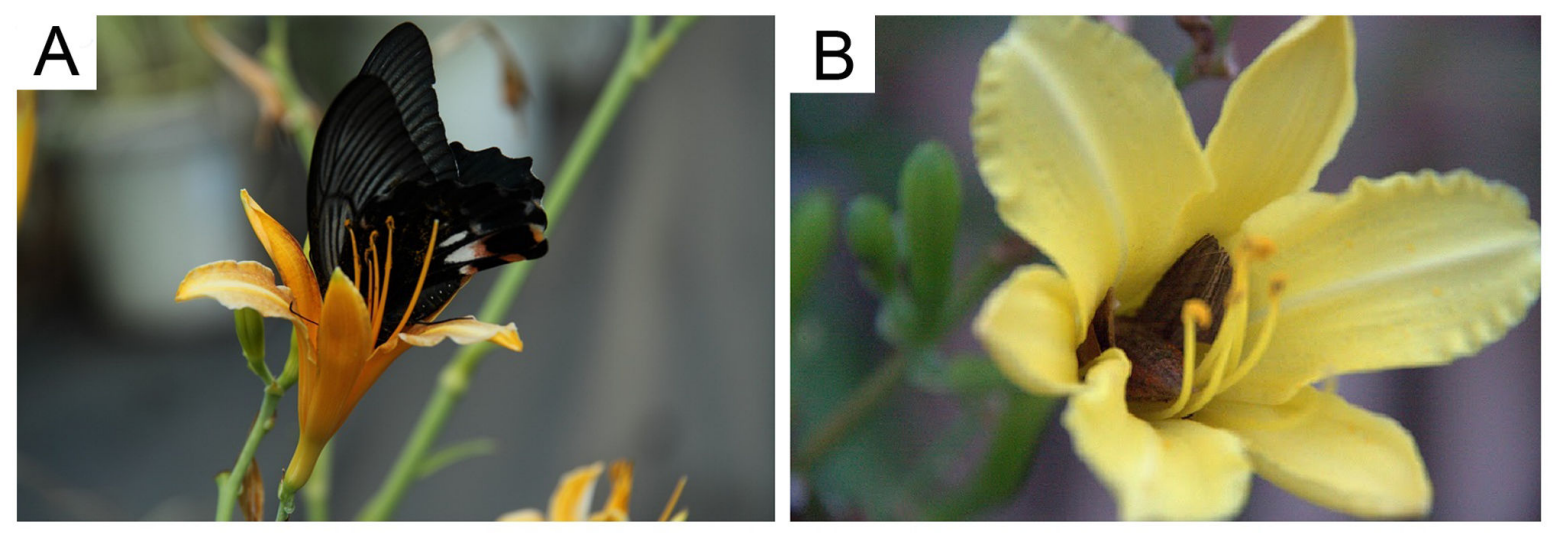
Flowers of *H. fulva* and F2 hybrid. (A) A swallowtail butterfly *Papilio xuthus* visiting a *H. fulva* flower. (B) A hawkmoth *Theretra japonica* visiting a F2 hybrid flower.

*H. cirina* is self-incompatible [31], and *H. fulva*, and their hybrids are also highly self-incompatible. *H. fulva* has diurnal, reddish flowers without recognizable scent [27], and the major pollinators are swallowtail butterflies, *Papilio* spp. (Figure 1A; [28]). *H. citrina* has nocturnal, yellowish flowers with a sweet scent [27], and the major pollinators are crepuscular and nocturnal hawkmoths, *Theretra* spp. (Figure 1B; [28]). To describe floral traits differences between two species, *H. citrina* and *H. fulva* used in our experiment, we scored flower color with the standard color chart (SCC, the Royal Horticultural Society, London, England) and floral scent intensity with a handheld odor meter (OMX-SR, Shinyei, Japan) (see Hirota et al. 2012 for details). Flower scent intensity was measured immediately after flower opening from 15 June to 26 August in 2006, from 19 July to 2 August in 2007, and from June 26 to August 4 in 2008. The measurements of scent intensity were performed for at least three flowers per plant and then averaged. We also measured corolla direction (angle to the vertical axis), height of the flower-bearing stem, and the anther-stigma distance (ASD) that can influence pollination process by swallowtail butterflies and/or nocturnal hawkmoths (Table S1 in File SI). 

For tepal color, the lower and higher SCC scores indicate more yellowish-colored and reddish-colored, respectively. SCC score of tepal color varied from 3 to 4 (3.2 ± 0.9) in *H. citrina* and from 21 to 23 (22.8 ± 0.1) in *H. fulva*. F2 hybrids showed high variability in tepal color from 3 to 23 (11.9 ± 0.31). Flower orientation was 41.4 ± 3.5 degree in *H. citrina* compared with 56.1 ± 1.3 degree in *H. fulva* (t test, t = 3.98, df = 16.62, P = 0.001), indicating that *H. citrina* had more horizontally oriented corolla. ASD of *H. citrina* was significantly shorter than that of *H. fulva* (t test, t = 6.48, P < 0.001), indicating that anthers of *H. fulva* were more exerted. ASD was correlated with style length (Pearson’s product-moment correlation; *H. fulva*, coefficient = 0.724, df = 36, *P* < 0.001; *H. citrina*, coefficient = 0.363, df = 29, *P* = 0.045), but not with stamen length (*H. fulva*, coefficient = 0.085, df = 36, *P* = 0.612; *H. citrina*, coefficient = -0.245, df = 29, *P* = 0.185). No significant difference was found in the number of pollen grains per anther between *H. fulva* and F2 hybrids (*H. fulva*, 1.203 × 10^4^, SD = 0.201 × 10^4^, n = 27; F2 hybrids, 1.269 × 10^4^, SD = 0.218 × 10^4^, n = 35; t test, t = 1.23, df = 58, *P* = 0.225). There was also no significant difference in the proportion of acetocarmine-stained pollen grains between *H. fulva* and F2 hybrids (*H. fulva*, 99.3%, n = 18; F2 hybrids, 99.0%, n = 27; glm with the binomial family and logit link, χ^2^ = -1.44, *P* = 0.230). Thus, outcross pollen acquisition and donation are good indicators of the maternal and paternal success.

### Design of experimental arrays

To examine the preference of pollinators and pollen movement, 24 and 12 potted plants of *H. fulva* and F2 hybrids were arranged randomly in a 6x6 square reticular pattern with a distance of 50 cm between each pot. By mixing 12 plants of F2 hybrids with 24 plants of *H. fulva*, our experiments mimicked the situations in which mutants for floral traits appeared in a lower frequency within a diurnal population like *H. fulva*. The mean values of floral traits in the experimental array are shown in Table S2 in File S1. We selected the plants and used microsatellite genotyping from single pollen grains for distinguish them by 10 microsatellite markers (Table S3 in File S1). In all experiments, we randomly selected one flower and cut off all remaining ones early in the morning if the plant had two or more flowers on an observation day. We replaced some of the 36 plants with new ones day by day because the longevity of a flower is only half a day, and each individual plant did not flower every day. Before starting our observation, we measured floral traits of experimental plants (tepal color by color chart, corolla direction, stem height, and ASD). The experimental array was placed inside the experimental field (Department of Biology, Kyushu University, Japan), where swallowtail butterflies and hawkmoths were common.

We conducted observations of pollinator visitation from 9:00 to 20:30 continuously, for 22 days from 18 July to 8 August in 2008. One observer watched experimental array and recorded pollinator visitation. Simultaneously, we used High-Definition Video Camera Recorder (XL H1, Canon, Japan) to record video images of an experimental array. We commenced the experiments each day from 9:30 and finished at 20:30. In *H. fulva*, the observed plants of *H. fulva* continued flowering until 20:30 because start-to-close time of *H. fulva* varies from 18:00 to 20:30 with a peak at 20:30 [27]. On the other hand, some plants of F2 hybrids started to close flowers in the afternoon because F2 hybrids have a wide range of start-to-close times [27]. We kept spare F2 hybrids that started to flower in the morning in an isolated house. Experimental plants that started to close flowering in the afternoon were replaced with spare ones still having an open flower. After sunset, we turned on a halogen lamp (500W) at a distance of 5m from the array to observe pollinators. This illumination does not affect color and scent preferences of hawkmoth [28].

We defined a trip of pollinator foraging as the process from the arrival of one pollinator to the experimental array to its departure from the array. After every trip, we collected 1 cm long pistils from the stigma of flowers that were visited by a pollinator and fixed them in 99.5% EtOH. Then, we replaced all plants that were visited by a pollinator with other plants having fresh stigma. Those plants were selected from spare stocks that started to flower in the morning and were kept in an isolated house. Such an experimental manipulation allowed pollinators to forage on fresh flowers in the experimental array that were not visited by the other pollinators. After the sunset, we cut off visited flowers instead of replacing whole plants because hawkmoth has limited time to showed high activity and we did not have enough time to replace them. Thus, in three out of eleven trip bouts, number of experimental flowers was fewer than 36.

### Treatment of pollen grains

We counted the number of pollen grains on each stigma under a stereomicroscope after immersing stigma in the fixative solution diluted with sterile distilled water. Then, 0.5µl of solution containing pollen grains was sucked by a micropipette and transferred it to a sterile distilled water drop placed on a glass slide. The individual pollen grains were washed repeatedly in sterile distilled water drops. During this step, the individual pollen grains were separated by moving them from one water drop to the next. Genotype of each pollen grain was determined by 10 microsatellite primers that were developed for *H. fulva* and *H. citrina* by Miyake and Yahara [32] and also from EST library of F1 hybrid between *H. fulva* and *H. citrina* by ourselves (Table S3 in File S1). When the number of pollen grains on a stigma was 72 or fewer, we isolated them to a single pollen grain and determined genotypes of all the pollen grains. If the number of pollen grains on a stigma exceeded 72 (approximately two times the number of experimental plants), we collected more than 72 pollen grains at random. 

### Single-pollen PCR amplification

PCR amplification of a single pollen grain was conducted following the method of Matsuki et al. [23] and Hasegawa et al. [33]. One pollen grain with 0.5µl of water was placed in a 0.2 ml PCR tube that contained 1.0µl of extraction buffer (0.01% SDS; 0.01% Proteinase K (TaKaRa); 0.01 M Tris-HCl, pH 7.8; 0.01 M EDTA) without any procedure to crush pollen wall, and incubated for 60 min at 54°C and heated for 10 min at 95 °C. The extract was used directly as a PCR template. PCR was performed in a GeneAmp PCR System 9600 (Applied Biosystems, Foster City, USA). The forward primers were labeled with fluorescent dye (G5 dye set: 6-FAM, VIC, NED or PET; Applied Biosystems) to simultaneously analysis ten microsatellite loci of similar allelic size and to avoid overlaps among loci with the same dye. Multiplex PCR amplification was carried out using a Multiplex PCR Kit (Qiagen KK) in a 10µl volume containing 1x Qiagen Multiplex PCR Master Mix, 0.2uM of each primer and 1.5µl of template extract from a pollen grain. We used the thermal cycler under the following cycle conditions: 94 °C for 15 min (hotstart), 38 cycles at 94°C for 30s, 54°C for 90s, 72°C for 1 min, and a final step at 60°C for 30 min. PCR product were electrophoresed on an ABI 3130xl Genetic Analyzer (Applied Biosystem), and allele sizes were determined with the fragment analysis software packages GeneScan 3.0 and GeneMapper (Applied Biosystem).

Pollen grains are haploids, but in some pollen samples, two or three alleles were found at one locus indicating that two or three pollen grains had been placed in one PCR tube and then amplified at the same time. We did not use these samples in the following analyses. As several loci were not amplified by pollen genotyping for several pollen grains, we used pollen samples that had more than seven and five genotyped loci for the following analysis of butterfly- and hawkmoth-pollination, respectively. We relaxed standards in hawkmoth-pollination because PCR efficiency of these samples was lower than PCR efficiency of the samples which were pollinated by butterflies. Finally, after PCR amplification, we performed paternity analysis for 828 out of 1758 butterfly-pollinated samples and 908 out of 1487 hawkmoth-pollinated samples. 

### Paternity analysis

For paternity analysis, 100 samples were examined by 10 microsatellite loci and the remaining 1636 samples were examined using nine or fewer microsatellite markers. Pollen grains were assigned as self if they did not contain non-maternal alleles. The paternity of each outcross pollen grain was assigned by a simple exclusion approach based on the multilocus genotypes of flowers that were visited by a pollinator before the pollen grain was deposited. If a pollen grain had two or more possible pollen donor candidates, we inferred paternity based on the maximum likelihood paternity assignment using the software CERVUS 2.0 [34]. For each pollen grain tested, the paternity likelihood of each candidate pollen donor was examined by the ratio of probabilities (the LOD score) that were calculated based on the multi-locus genotype of a tested pollen grain, the multi-locus genotypes of candidate pollen donors, and the population allele frequencies [35]. Confidence levels are determined through simulations and defined by the statistic delta (Δ) [34] where Δ is the difference between the LOD scores of the most likely male and the second most likely male. Significance tests for each assignment were conducted using computer simulations, running 10,000 iterations with the 95% strict confidence level and the 80% relaxed confidence level. In detail, parameters used for simulation analyses in CERVUS were the following: 10,000 cycles; the number (n = 473) of all individuals in the experimental array; 90% as the proportion of candidate parents sampled; 100% as the proportion of the loci typed; a typing error rate of 1%; and a confidence level of 80%. When the significance of the paternity analysis was less than 80%, we excluded those samples from subsequent analysis. As a result, paternity of 1736 pollen grains was identified among 3245 pollen grains collected from 151 flowers and subjected to PCR.

### Pollination success

Four indicators were used to evaluate pollination success per flower per trip bout. We used the number of visits within a trip bout as an indicator of attraction. To evaluate the efficiency of pollen transfer, we used outcross pollen acquisition per flower per trip bout and pollen donation per flower per trip bout. Number of outcross pollen grains acquired within a trip bout was used as an indicator of maternal success, because both *H. fulva* and *H. citrina* are highly self-incompatible [36]. Number of pollen grains donated to other plants within a trip bout was used as an indicator of paternal success. To combined attraction and efficiency of maternal and paternal success, we calculated “combined pollination success” *W* based on the following equation.

*W = X + Y × (Ave. X / Ave. Y),*

where *X* is the number of outcross pollen grains acquired, *Y* is the number of pollen grains donated to other plants, *Ave.X* and *Ave.Y* represent the average of *X* and *Y*, respectively.

We also calculated *W* under no competition by assuming Ave. X / Ave. Y = 1. This case is expected when pollen grains deposited on a stigma are fewer than ovules and most of the deposited outcross pollen grains are successful in fertilizing ovules.

### Statistical analysis

To examine the effects of tepal color, scent intensity and floral morphology on visitation, outcross pollen acquisition and donation, and combined pollination success, we developed hierarchical Bayesian models. The response variables of the model were the number of visitations per flower per trip bout, outcross pollen acquisition, pollen donation and combined pollination success. Tepal color, scent intensity, corolla direction, stem height and ASD as well as the quadratic components tepal color^2^, scent intensity^2^, corolla direction^2^, stem height^2^ and ASD^2^ were included as the explanatory variables. The quadratic terms allow us to explore the possibility of stabilizing or disruptive selection. We excluded interaction term to models to evaluate the direct effect of each trait on the dependent variable. If the 95% CI for a regression coefficient included zero, we classified the regression coefficient into the [no effect] group. Otherwise, the effects of regression coefficients were classified into [negative] and [positive] groups according to the sign of the median of posterior distributions of each regression coefficient. When a quadratic regression coefficient was negative, a trait value of the maximum success was calculated by *z* ^*^ = -β/2γ. If the 95% confidence intervals of *z* ^*^ was within the phenotypic value, it indicated whether the trait was under stabilizing or disruptive selection depending on the sign of *z* ^*^. For better convergence in parameter estimation, all explanatory variables were standardized to mean = 0 and SD = 1 and all measures of pollination success except visitations, were standardized to SD = 1. Trip bout ID and genet ID of the experimental plants were added as random effects. 

First, to investigate the selection on floral traits through visitation, we assumed that the visitation followed a Poisson distribution with mean λ, where λ represents the mean number of visitations per flower per trip bout of experimental plants. The model structures of the analysis of visitation were as follows:

Visitations ~ Poisson (λ),
log λ = α
+ β_1_ × (tepal color) + γ_1_ × (tepal color)^2^
+ β_2_ × (scent intensity) + γ_2_ × (scent intensity)^2^
+ β_3_ × (corolla direction) + γ_3_ × (corolla direction)^2^
+ β_4_ × (stem height) + γ_4_ × (stem height)^2^
+ β_5_ × (ASD) + γ_5_ × (ASD)^2^
+ r[i] × (trip bout ID)
+ rp[i] × (genet ID of experimental flowers)

where α is the intercept, β and γ represent the linear and quadratic coefficients of explanatory variables, respectively, and r and rp represent the random effects of individual pollinators and individual plants, respectively. Prior distributions of these parameters are listed in Table S4 in File S1. 

Second, to investigate the selection on floral traits through outcross pollen acquisition and pollen donation, we assumed that the number of outcross pollen acquisition and the number of pollen donation followed a Poisson distribution of mean λ. In this analysis, to measure efficiency of outcross pollen acquisition and donation par single visit, we excluded unvisited flowers from data set, and added the number of visits per flower per trip bout to explanatory variables as a fixed effect.

Number of pollen grains ~ Poisson (λ),
log λ = α
+ β_1_ × (tepal color) + γ_1_ × (tepal color)^2^
+ β_2_ × (scent intensity) + γ_2_ × (scent intensity)^2^
+ β_3_ × (corolla direction) + γ_3_ × (corolla direction)^2^
+ β_4_ × (stem height) + γ_4_ × (stem height)^2^
+ β_5_ × (ASD) + γ_5_ × (ASD)^2^
+ β_6_ × (number of visits per flower per trip bout)
+ r[i] × (trip bout ID)
+ rp[i] × (genet ID of experimental flowers)

Where α is the intercept, β and γ represent the linear and quadratic coefficients of explanatory variables, respectively, and r and rp represent the random effects of individual pollinators and individual plants, respectively.

Finally, to investigate the selection on floral traits through the combined pollination success, we assumed that the combined pollination success followed a Poisson distribution of mean λ. α, βs, r and rp are the same as above.

Combined pollination success ~ Poisson (λ),
log λ = α
+ β_1_ × (tepal color) + γ_1_ × (tepal color)^2^
+ β_2_ × (scent intensity) + γ_2_ × (scent intensity)^2^
+ β_3_ × (corolla direction) + γ_3_ × (corolla direction)^2^
+ β_4_ × (stem height) + γ_4_ × (stem height)^2^
+ β_5_ × (ASD) + γ_5_ × (ASD)^2^
+ r[i] × (trip bout ID)
+ rp[i] × (genet ID of experimental flowers)

Parameter estimation, or the sampling of posterior distributions, was analyzed using the Markov chain Monte Carlo (MCMC) method with WinBUGS 1.4.3 [37] and the R2WinBUGS package [38] on R 2.13.1. The non-informative priors for fixed-effect parameters are Gaussian distributions, and those of random-effect parameters are Gaussian distributions of mean 0 and standard deviation τ. The variance parameter τ is referred to as a hyperparameter of which the prior distributions are noninformative uniform distributions, 0 < τ < 10^4^. Prior distributions of these parameters are listed in Table S4 in File S1. The posterior samples were obtained from three independent Markov chains in which 3000 values were sampled with 20 iteration intervals after a burn-in of 10000 iterations. The convergence of the Markov chains was checked with Ȓ [39] for each parameter. The Ȓ values obtained were less than 1.1 for all parameters. The median and 95% Bayesian confidence interval (CI) for each parameter were evaluated using the MCMC samples. All source code lists for the analysis were written in R and BUGS languages.

## Results

### Pollinator attraction

Flowers visited by swallowtail butterflies or hawkmoths showed high variability in tepal color, floral scent, corolla direction, stem height and anther-stigma distance (ASD), likely reflecting the segregation in F2 hybrids (Table S5 in File S1). During the experiment, we observed 8 trips of swallowtail butterflies (*Papilio memnon*) and 11 trips of hawkmoths (*Theretra japonica*, *T. oldenlandiae* and *T. silhetensis silhetensis*). Swallowtail butterflies visited 63 flowers (58 flowers of *H. fulva* and 5 flowers of F2 hybrids out of 288 experimental flowers) and hawkmoths visited 88 flowers (75 flowers of *H. fulva* and 13 flowers of F2 hybrids out of 348 experimental flowers). Swallowtail butterflies visited a flower twice within a trip bout for 13 flowers and a flower only once for other 50 flowers. Hawkmoths visited a flower twice within a trip bout for 13 flowers, and a flower only once for other 75 flowers. Thus, the number of visits per flower per trip bout varied from zero to two both in swallowtail butterflies and in hawkmoths. The number of visits of swallowtail butterflies within a trip bout (0, 1 or 2) significantly varied with floral scent intensity and stem height: the quadratic regression coefficient for floral scent intensity was significantly negative (γ ± SD -3.842 ± 1.909, lower CI = −8.312, upper CI = -1.015). The linear and quadratic regression coefficient for stem length was significantly positive (β ± SD 0.452 ± 0.183, lower CI = 0.088, upper CI = 0.822) and negative (γ ± SD -0.268 ± 0.143, lower CI = −0.569, upper CI = -0.013) (Table 1), respectively (Table 1). The maximum success for scent intensity (*z* ^*^ = -0.217, lower CI= -0.430, upper CI = 0.150) was within the range of phenotypic values in experimental population (-0.689 to 3.458), indicating that scent intensity was under stabilizing selection. The maximum success for stem height (*z* ^*^ = 0.843, lower CI= -0.037, upper CI = 5.143) exceeded the range of phenotypic values (-3.024 to 2.638). In this case, we cannot distinguish whether the stem height under stabilizing selection or directional selection. The number of visits of hawkmoths within a trip bout varied significantly with tepal color: linear regression coefficient for tepal color (β ± SD 1.150 ± 0.411, lower CI = 0.358, upper CI = 1.970) was significantly positive. This indicates that hawkmoths preferred reddish flowers.

**Table 1:** Means and 95% confidence intervals of the posterior distribution of linear (β) and quadratic (γ) parameters in analyses of attraction.

	**β**				**γ**			
**Traits**	**Mean**	**SD**	**2.5%**	**97.5%**	**Mean**	**SD**	**2.5%**	**97.5%**
**Swallowtail butterflies**								
Tepal color	0.698	0.523	-0.274	1.778	-0.461	0.505	-1.518	0.470
**Scent intensity**	-1.665	1.446	-5.140	0.521	**-3.842**	**1.909**	**-8.312**	**-1.015**
Corolla direction	-0.020	0.172	-0.373	0.312	-0.211	0.140	-0.517	0.022
**Stem height**	**0.452**	**0.183**	**0.088**	**0.822**	**-0.268**	**0.143**	**-0.569**	**-0.013**
ASD	-0.043	0.163	-0.359	0.291	0.000	0.089	-0.359	0.159
**Hawkmoths**								
**Tepal color**	**1.150**	**0.411**	**0.358**	**1.970**	0.259	0.277	-0.287	0.824
Scent intensity	0.714	0.381	-0.023	1.453	-0.288	0.162	-0.617	0.008
Corolla direction	0.155	0.151	-0.135	0.460	-0.202	0.120	-0.459	0.008
Stem height	-0.120	0.138	-0.400	0.152	0.044	0.074	-0.113	0.177
ASD	0.145	0.159	-0.299	0.465	-0.090	0.100	-0.299	0.101

Bold values are the effects of the parameters classified into groups of [negative] or [positive].

### Pollen acquisition and pollen donation success

**Figure 2:**
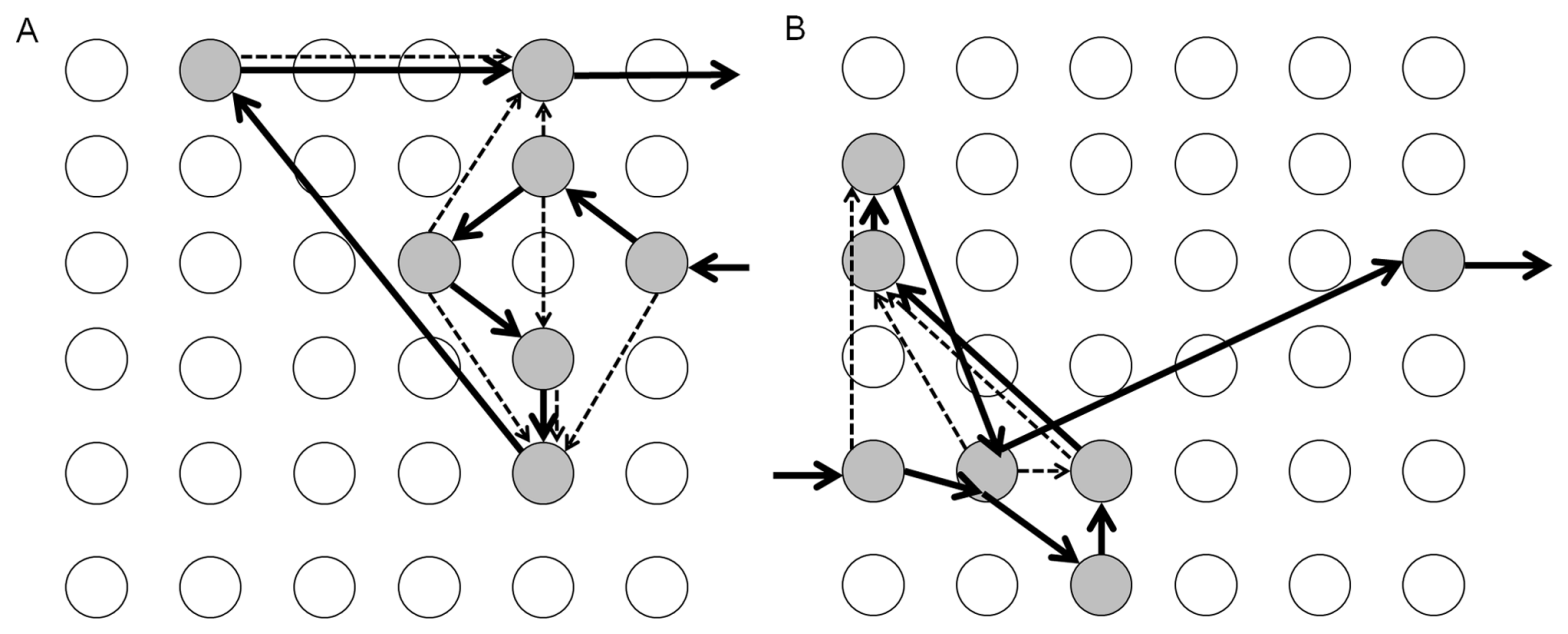
Examples of foraging bouts and pollen movement. Each circle indicates experimental flowers. Solid arrows indicate foraging movements of swallowtail butterfly (A) and hawkmoth and gray circles are visited flowers. Dashed arrows show pollen movement from donors to recipients.

We determined paternity of 1736 pollen grains including 1129 self pollen grains and 607 outcross pollen grains. Paternity data of outcross pollen enabled us to trace the movement of pollen grains carried by a swallowtail butterfly or a hawkmoth. Figure 2 shows an example of foraging bout and pollen movement by a butterfly or a hawkmoth. Pollen grains removed by a pollinator were not always deposited on the next flower, but sometimes on the other flowers visited later. Our analyses provide evidence of selection for tepal color, scent intensity, and ASD (in the outcross pollen acquisition) by swallowtail butterflies as well as selection for scent intensity (in the pollen donation) and ASD (in the outcross pollen acquisition) by hawkmoths (Table 2). Effects of those traits are described below in detail.

**Table 2:** The summarizing results of the hierarchical Bayesian analyses.

	**Attraction**	**Efficiency**		**Combined**
**Traits**		**Pollen acquisition**	**Pollen donation**	
**Swallowtail butterflies**				
Tepal color	ns	stabilizing	ns	ns
Scent intensity	stabilizing	stabilizing	ns	stabilizing
Corolla direction	ns	ns	ns	stabilizing
Stem height	stabilizing or directional to be taller	ns	ns	ns
ASD	ns	directional to be smaller	ns	disruptive
**Hawkmoths**				
Tepal color	directional	ns	ns	ns
Scent intensity	ns	ns	stabilizing or directional to be weaker	ns
Corolla direction	ns	ns	ns	ns
Stem height	ns	ns	ns	ns
ASD	ns	directional to be smaller	ns	ns

Directional, stabilizing and disruptive selections were classified by 95% CI of confidence intervals of the posterior distribution of linear (β), quadratic (γ) parameters and maximum/minimum success (*z* ^*^ = -β/2γ).

In butterfly-mediated pollination, the number of outcross pollen acquired varied significantly with tepal color, scent intensity and ASD (Table 3). For tepal color, the linear regression coefficient was significantly negative (β ± SD -3.177 ± 1.674, lower CI = -6.689, upper CI = -0.110) and the quadratic regression coefficient was also significantly negative (γ ± SD -1.799 ± 0.859, lower CI = -3.662, upper CI = -0.237). For scent intensity, the quadratic regression coefficient was significantly negative (γ ± SD -1.146 ± 0.662, lower CI = −2.646, upper CI = -0.044). For ASD, the linear regression coefficient was significantly negative (β ± SD -0.820 ± 0.369, lower CI = -1.637, upper CI = -0.171). The maximum success for tepal color (*z* ^*^ = -0.883, lower CI= -1.666, upper CI = -0.190) and for scent intensity (*z* ^*^ = 0.409, lower CI= -0.297, upper CI = 2.496) were within the range of phenotypic values (tepal color: -5.380 to 0.359, scent intensity: -1.259 to 2.588),indicating that both traits were under stabilizing selection. 

**Table 3:** Means and 95% confidence intervals of the posterior distribution of linear (β) and quadratic (γ) parameters in analyses of outcross pollen acquisition.

	**β**				**γ**			
**Traits**	**Mean**	**SD**	**2.5%**	**97.5%**	**Mean**	**SD**	**2.5%**	**97.5%**
**Swallowtail butterflies**								
**Tepal color**	**-3.177**	**1.674**	**-6.689**	**-0.110**	**-1.799**	**0.859**	**-3.662**	**-0.237**
**Scent intensity**	0.938	0.536	-0.095	2.035	**-1.146**	**0.662**	**-2.646**	**-0.044**
Corolla direction	-0.473	0.483	-1.460	0.486	-0.675	0.435	-1.639	0.050
Stem height	0.182	0.421	-0.639	1.015	-0.285	0.386	-1.116	0.420
**ASD**	**-0.820**	**0.369**	**-1.637**	**-0.171**	-0.116	0.487	-1.150	0.700
Visits	1.004	0.773	-0.451	2.511				
**Hawkmoths**								
Tepal color	-0.682	1.045	-2.772	1.390	-0.111	0.264	-0.649	0.377
Scent intensity	-1.362	0.969	-3.538	0.406	0.100	0.247	-0.421	0.582
Corolla direction	-0.313	0.402	-1.105	0.480	-0.141	0.295	-0.746	0.418
Stem height	-0.325	0.493	-1.343	0.587	-0.666	0.402	-1.528	0.018
**ASD**	**-1.164**	**0.398**	**-2.044**	**-0.479**	0.018	0.367	-0.755	0.734
Visits	-2.181	1.996	-6.829	1.043				

63 and 88 experimental flowers were visited by swallowtail butterflies and hawkmoths, respectively. These flowers were analyzed to evaluate the selection on efficiency of outcross pollen acquisition. Bold values are the effects of the parameters classified into groups of [negative] or [positive].

In hawkmoth-mediated pollination, the number of outcross pollen grains acquired was only influenced by ASD: the linear regression coefficient was significantly negative (β ± SD -1.164 ± 0.398, lower CI = -2.044, upper CI = -0.479, Table 3). The number of pollen grains donated varied significantly with the scent intensity and the number of visits on the flower. The quadratic regression coefficient for scent intensity was significantly negative (γ ± SD -2.507 ± 1.749, lower CI = −6.661, upper CI = -0.129, Table 4). The maximum success (*z* ^*^ = -0.036, lower CI= -1.821, upper CI =1.370) was within the range of phenotypic values (-0.643 to 5.672), though the lower CI of maximum success exceeded the range of phenotypic values. 

**Table 4:** Means and 95% confidence intervals of the posterior distribution of linear (β) and quadratic (γ) parameters in analyses of pollen donation.

	**β**				**γ**			
**Traits**	**Mean**	**SD**	**2.5%**	**97.5%**	**Mean**	**SD**	**2.5%**	**97.5%**
**Swallowtail butterflies**								
Tepal color	0.479	2.293	-4.588	4.733	-0.223	0.945	-2.427	1.319
Scent intensity	-0.050	0.871	-1.833	1.768	-0.559	0.988	-2.889	1.022
Corolla direction	-0.023	0.587	-1.165	1.167	-0.342	0.498	-1.380	0.602
Stem height	0.537	0.590	-0.597	1.731	0.007	0.383	-0.784	0.723
ASD	-0.153	0.467	-1.140	0.715	0.709	0.419	-0.064	1.635
Visits	1.252	1.073	-0.658	3.604				
**Hawkmoths**								
Tepal color	0.003	1.890	-4.075	3.465	-0.316	1.227	-3.045	1.718
**Scent intensity**	-0.182	1.332	-2.952	2.277	**-2.507**	**1.749**	**-6.661**	**-0.129**
Corolla direction	-0.115	0.375	-0.837	0.623	0.020	0.258	-0.525	0.511
Stem height	0.776	0.522	-0.087	1.910	-0.434	0.327	-1.126	0.154
ASD	0.673	0.432	-0.084	1.584	-0.164	0.281	-0.720	0.379
**Visits**	**1.643**	**0.656**	**0.430**	**3.019**				

63 and 88 experimental flowers were visited by swallowtail butterflies and hawkmoths, respectively. These flowers were analyzed to evaluate the selection on efficiency of pollen donation. Bold values are the effects of the parameters classified into groups of [negative] or [positive].

### Total pollination success

The total pollination success was quantified by combining the maternal and paternal success, assuming pollen competition (Ave. X / Ave. Y is not unity) or no pollen competition (Ave. X / Ave. Y = 1). The observed value of Ave. X / Ave. Y was 1.87 in butterfly-mediated pollination and 1.06 in hawkmoth-mediated pollination. Both analyses gave similar results. The results from the former analysis are described below and from the latter analyses are shown in Table S6 in File S1. 

The combined pollination success varied with scent intensity, corolla direction and ASD (Table 5). The quadratic regression coefficient was significantly negative for scent intensity (γ ± SD -21.111 ± 11.619, lower CI = -49.508, upper CI = -3.828, Table 5) and for corolla direction (γ ± SD -1.253 ± 0.362, lower CI = −2.029, upper CI = -0.612). The maximum success for scent intensity (*z* ^*^ = -0.338, lower CI= -0.462, upper CI = 0.019) and for corolla direction (*z* ^*^ = 0.001, lower CI= -0.352, upper CI = 0.241) were within the range of phenotypic values (scent intensity: -0.689 to 3.458, corolla direction: -3.751 to 1.559), indicating that both traits are under stabilizing selection. For ASD, the linear regression coefficient was significantly negative (β ± SD -0.678 ± 0.240, lower CI = -1.083, upper CI = -0.303) and the quadratic regression coefficient was significantly positive (γ ± SD 0.339 ± 0.123, lower CI = 0.098, upper CI = 0.591). The minimum success for ASD (*z* ^*^ = 1.000, lower CI= 0.222, upper CI = 2.766) was within the range of phenotypic values (-2.201 to 4.386), indicating that ASD is under disruptive selection. In hawkmoth-mediated pollination, we did not find any traits with significant effects on the combined pollination success using the regression analyses.

**Table 5:** Means and 95% confidence intervals of the posterior distribution of linear (β) and quadratic (γ) parameters in analyses of combined pollination success calculated with the observed Ave. X/Ave. Y.

	**β**				**γ**			
**Traits**	**Mean**	**SD**	**2.5%**	**97.5%**	**Mean**	**SD**	**2.5%**	**97.5%**
**Swallowtail butterflies**								
Tepal color	2.157	1.815	-1.145	6.099	-2.999	2.832	-9.876	1.186
**Scent intensity**	-14.286	9.937	-38.232	0.178	**-21.111**	**11.619**	**-49.508**	**-3.828**
**Corolla direction**	0.002	0.335	-0.630	0.685	**-1.253**	**0.362**	**-2.029**	**-0.612**
Stem height	0.386	0.292	-0.174	0.945	0.028	0.240	-0.472	0.488
**ASD**	**-0.678**	**0.240**	**-1.083**	**-0.303**	**0.339**	**0.123**	**0.098**	**0.591**
**Hawkmoths**								
Tepal color	1.357	0.840	-0.15	3.166	0.187	0.554	-0.887	1.262
Scent intensity	-0.124	0.802	-1.676	1.417	-0.192	0.355	-0.956	0.463
Corolla direction	-0.271	0.226	-0.701	0.176	-0.268	0.188	-0.669	0.056
Stem height	0.041	0.266	-0.471	0.572	-0.244	0.193	-0.633	0.114
ASD	-0.208	0.216	-0.619	0.217	0.021	0.158	-0.304	0.321

All experimental flowers (swallowtail butterfly-pollination, N = 288, hawkmoth-pollination, N = 248) were analyzed to evaluate the selection through the combined pollination success. Bold values are the effects of the parameters classified into groups of [negative] or [positive].

In summary, we found evidence of significant selection for all five traits in at least one of the four measures; the number of pollinator visits per flower, the number of pollen grains acquired, the number of pollen grains donated, and the combined pollination success (Table 2). However, the traits had no significant effects on all four measures and only one trait (scent intensity) had consistently significant effects on three measures (the number of pollinator visits per flower, the number of pollen grains acquired, and the combined pollination success) in butterfly-mediated pollination. We found evidence of selection for all four measures; in three cases for the number of pollinator visits per flower, four cases for the number of pollen grains acquired (maternal success), one case for the number of pollen grains donated (paternal success), and three cases for the combined pollination success. Among three significant cases for the combined pollination success, only one case (scent intensity in butterfly-mediated pollination) was significant in either maternal or paternal success. We found no evidence for selection by hawkmoths on the combined pollination success.

## Discussion

This is the first study of pollinator-mediated selection using single-pollen genotyping. As for the materials, this study follows two pioneering works that provided remarkable opportunities through fertile hybrids of sister species with divergent pollination syndromes: *Mimulus cardinalis* vs *M. lewsii* [1,40] and *Petunia integrifolia* or *P. exserta* vs *P. axillaris* [24,25]. By determining paternity of individual pollen grains deposited on stigmas by swallowtail butterflies and hawkmoths in an experimental array of *H. fulva* (a swallowtail butterflies-pollinated species) mixed with F2 hybrids of *H. fulva* and *H. citrina* (a hawkmoths-pollinated species), we aimed at detecting swallowtail butterflies- and hawkmoths-mediated selection for flower color, scent intensity and mechanical traits. The findings include some results that are expected and some that may not expected.

Swallowtail butterflies imposed selection on reddish color and weak scent. The number of outcross pollen grains acquired is a quadratic function of flower color with the maximum at -0.883, while flower color varied from -5.380 (most yellowish) to 0.359 (most reddish). Thus, reddish color is favored by swallowtail butterflies. Although butterflies visited *H. fulva* flowers 11.6 times more often than F2 hybrids, flower color did not significantly affect on the number of butterfly visits per flower, the number of pollen grains donated and the combined pollination success. In our previous study, swallowtail butterflies significantly preferred to visit reddish flowers [28]. This difference could be partly due to the visitation model (in experimental array) which included morphological traits and the quadratic explanatory variables.

The most convincing evidence for butterfly-mediated selection on weak scent is the significant effect of scent intensity and also the evidence of stabilizing selection on three measures (the number of butterfly visits per flower, the number of outcross pollen acquired and the combined pollination success). The combined pollination success was maximal at -0.338 while scent intensity varied from -0.689 to 3.458 in our experimental plants. Thus, weak scent (almost unrecognizable for human) was favored by swallowtail butterflies. This finding explains why *H. fulva*, with no recognizable scent, is mainly pollinated by swallowtail butterflies.

One of the most unexpected results from this study was that hawkmoths showed significant preference for reddish flower color. This finding is incongruent with previous result that hawkmoths preferred yellowish flowers of *Hemerocallis* [28]. This incongruency might be due to the experimental design. In previous study [28], our observations on the same experimental plants were recorded from morning till night. For the present study, we replaced all plants visited by a butterfly or a hawkmoth with new ones after every trip bout due to stigma collection. As a result, all plants in the experimental array had full nectar in their flower tubes. Experimental plants in the previous study, however, should have had a reduced amount of nectar after pollinator visits. In particular, most reddish flowers were visited by diurnal pollinators and thus nectar of those flowers might have depleted during foraging trip of diurnal pollinators. For hawkmoths, therefore, it would be a better strategy not to forage on depleted reddish flowers but upon undepleted yellowish flowers. Because reddish flowers were more abundant in the experimental array of this study, hawkmoths were likely to learn the association of red color with nectar availability. It is well documented that hawkmoths can learn the association of tepal color with the presence of nectar rewards [41]. 

While hawkmoths significantly preferred reddish flowers, we did not find any statistically significant effect of flower color on the combined pollination success during hawkmoth visits. This lack of effect of flower color might be partly due to the fact that only 13 flowers of *H. fulva* were visited twice, and thereby increase the probability of success of pollen donation. Although the reason of the lack of significant effects of flower color on the combined pollination success remains unclear, it is likely that hawkmoths are rather opportunistic pollinators and can impose only weak selection on flower color, if any.

A second unexpected finding is evidence of selection for weaker scent by hawkmoths: the number of pollen grains donated is a quadratic function of scent intensity with the maximum at -0.036 (lower CI = -1.821, upper CI = 1.370) while scent intensity varied from -0.643 (weakest) to 5.672 (strongest) though the lower CI exceeded the phenotypic range. Thus, scent intensity is under stabilizing selection on near-zero emission or directional selection for weaker scent. The efficiency of pollen donation might be affected by differences in the feeding behavior of hawkmoths (depending on scent intensity). However, scent intensity did not give any significant effect on the combined pollination success. Hawkmoths can impose only weak selection on floral scent intensity at best.

The third unexpected finding in this study is the evidence of directional selection toward shorter ASD. We obtained this evidence for the number of outcross pollen acquired in both butterfly- and hawkmoth-mediated pollination (Table 3). This finding was inconsistent with our earlier expectation that larger and smaller ASD may be favored by swallowtail butterflies and hawkmoths, respectively because *H. fulva* has larger ASD than *H. citrina*. ASD was strongly correlated with style length. This finding means that shorter style is favored by both pollinators. The shorter style probably assures the pollen deposition [42]. In *Hemerocallis* species, both butterflies and hawkmoths crawl into the funnel-shaped tepals (Figure 1). During this process, an exserted pistil often could be touched bythe under surface of the wings of butterflies or the body and wings of hawkmoths. The long exserted pistil may have lower chance to touch by the body and/or wings of pollinators than the short-exserted pistil.

The present findings do not support the hypothesis that yellow flower color and strong scent intensity, the distinctive floral characteristics of *H. citrina* having evolved as adaptations to hawkmoths [28,43]. Based on these findings, we suggest that the key trait that triggers the evolution of nocturnal flowers may be flowering time rather than flower color and scent. Flowering time is known to be regulated by major genes [44,45] and a single gene mutation can shift the timing of flower opening from morning to evening [27]. Thus, if the availability of diurnal pollinators was reduced, the nocturnal, reddish, unscented flowers variants were probably pollinated by nocturnal pollinators including both hawkmoths and other generalists. Hence, the evolution of flower color and scent may be followed by the fixation of the nocturnally flowering variants. Based on these findings, we suggest two new hypotheses for the evolution of yellow flower color and strong scent intensity in nocturnal flowers*.*

First, the evolution of yellow flower color may have been driven by abiotic factors at night. While pollinators have been considered to play a dominating role in floral evolution, increasing evidence revealed significant influences of abiotic factors upon the evolution of floral traits (reviewed in 46). For example, anthocyanins, associated with reddish flower color of *H*. *fulva* [47] and many other plants [40,48], have some important functions in plant physiology rather than attracting pollinators. First, anthocyanins absorb radiation in the ultraviolet (UV) region of the spectrum and protect plant cells from the induction of damage caused by UV radiation [49]. Second, anthocyanins pigmented flowers often better tolerate stressful conditions like drought and heat than anthocyanin-less morphs [50,51]. Thus, the floral anthocyanins are strongly favored under daytime environment. In contrast, both UV radiation and water or heat stress are considered to be weaker at night than daytime, and thus floral anthocyanin production might be too costly at night. Release from UV damage and water/heat stress may account for the loss of anthocyanins in *H. citrina* and nocturnal flowers in general.

The second hypothesis is that strong scent intensity may have evolved as an adaptation to nocturnal pollinators, but not to hawkmoths. Other studies showed that floral scent is not only associated with hawkmoths but also with other nocturnal pollinators, such as noctuid moths [52,53] and beetles [54]. Hence, it seems reasonable to speculate that when the availability of swallowtail butterflies was reduced in an unscented flower-dominated ancestral population, floral scent emission variant in a population might have been favored in attracting generalist nocturnal pollinators. Consequently, the evolution of floral scent is thought to have been driven by generalist nocturnal pollinators rather than by hawkmoths. 

The results of this study raise questions about the traditional hypothesis that flower color and/or floral scent in night flowering species have evolved in response to selection by hawkmoths. Hawkmoth is one of the conspicuous nocturnal pollinators having relatively large body size and long proboscis. Thus, nocturnal hawkmoths have been studied intensively as a selective agent on floral traits [24,55]. However, it is likely that hawkmoths are opportunistic pollinators and can impose only weak selection on flower color and scent. To understand the evolutionary process of flower color and scent changes in nocturnal flowers, further experiments are needed to determine the role of generalist pollinators. For those experiments, *Hemerocallis* provides a hopeful model system [56] and single-pollen genotyping provides a powerful tool for the quantification female and male components of pollination success.

## Supporting Information

File S1 **Tables S1-S6. Table S1:** Floral traits of H. fulva, H. citrina and F2 hybrids. **Table S2:** Floral traits of flowers used in observations of swallowtail butterflies or hawkmoths. **Table S3:** Characteristics of the microsatellite loci. **Table S4:** Definitions of prior distribution of parameters. **Table S5:** Floral traits of flowers visited by swallowtail butterflies or hawkmoths. **Table S6:** Means and 95% confidence intervals of the posterior distribution of parameters in analyses of combined fitness index calculating with Ave. X/Ave. Y = 1.
(PDF)
