# Correction: Pollinator-Mediated Selection on Flower Color, Flower Scent and Flower Morphology of *Hemerocallis*: Evidence from Genotyping Individual Pollen Grains On the Stigma

**DOI:** 10.1371/journal.pone.0117885

**Published:** 2015-02-10

**Authors:** The PLOS ONE Staff

There is an error in the legend for Figure 1, “Flowers of *H*. *fulva* and F2 hybrid.” The complete, correct Figure 1 legend is:

**Figure 1. Flowers of *H*. *fulva* and F2 hybrid**. (A) A swallowtail butterfly *Papilio memnon* visiting a *H*. *fulva* flower. (B) A hawkmoth*Theretra japonica* visiting a F2 hybrid flower.
