# Supplementary material for: Pollinator-Mediated Selection on Flower Color, Flower Scent and Flower Morphology of Hemerocallis: Evidence from Genotyping Individual Pollen Grains On the Stigma
Source: PLoS One. 2013 Dec 23;8(12):e85601. doi: 10.1371/journal.pone.0085601 (PMC3871637; doi:10.1371/journal.pone.0085601)
Supplement: File S1 — Tables S1-S6. Table S1: Floral traits of H. fulva, H. citrina and F2 hybrids. Table S2: Floral traits of flowers used in observations of swallowtail butterflies or hawkmoths. Table S3: Characteristics of the microsatellite loci. Table S4: Definitions of prior distribution of parameters. Table S5: Floral traits of flowers visited by swallowtail butterflies or hawkmoths. Table S6: Means and 95% confidence intervals of the posterior distribution of parameters in analyses of combined fitness index calculating with Ave. X/Ave. Y = 1. (PDF) [file pone.0085601.s001.pdf]

Supporting information for

Pollinator-mediated selection on flower color, flower scent and flower morphology of *Hemerocallis*: evidence from genotyping individual pollen grains on the stigma

S. K. Hirota, K. Nitta, Y. Suyama, N. Kawakubo, A. A. Yasumoto, T. Yahara

Table S1 Floral traits of *H. fulva*, *H. citrina* and F2 hybrids.

Trait	*H. fulva*					*H. citrina*					F2 hybrids				
	mean	se	min.	max.	N	mean	se	min.	max.	N	mean	se	min.	max.	N
Tepal color (color chart)	22.8	0.10	21	23	50	3.2	0.87	3	4	8	11.9	0.31	3	23	269
Scent intensity	2.3	0.28	0.1	7.3	49	21.2	2.39	11.3	33.3	3	12.4	0.57	0.2	49.0	119
Corolla direction (°)	56.1	1.28	28.9	79.3	48	41.4	3.49	3.0	57.7	13	54.9	1.10	8.7	139.3	106
Stem height (cm)	93.8	1.93	73.5	134.6	41	92.2	4.01	68.0	112.7	8	105.3	1.10	64.5	142.8	259
ASD (mm)	22.5	0.92	12.6	39.4	41	13.2	1.12	6.0	20.0	14	14.1	0.51	0.5	32.2	143

Table S2 Floral traits of flowers used in observations of swallowtail butterflies or hawkmoths

Floral traits	Observation of swallowtail butterflies			Observation of hawkmoths		
	Mean (SE)	Min.	Max.	Mean (SE)	Min.	Max.
Tepal color	18.6 (0.40)	3	23	18.7 (0.35)	3	23
Floral scent	5.61 (0.47)	0.1	33.3	5.29 (0.42)	0.1	33.3
Corolla Direction (°)	64.7 (0.95)	4	90	67.8 (0.76)	4	90
Stem height (cm)	98.9 (0.89)	53	139	99.2 (0.80)	60	132
ASD (mm)	21.4 (0.66)	0	70.5	20.3 (0.55)	0	56.4

Table S3 Characteristics of the microsatellite loci

Locus	Repeat Motif	Primer sequences (5'-3')	Reference
HemC4	(CA)24	F: TGGGTCGCATAAAATGTGAA	Miyake & Yahara (2006)
		R: AAGTGCACAACGGAAGGTTT	
HemC10	(AC)20	F: GCAGCCATAAATCCCACAAC	Miyake & Yahara (2006)
		R: CGTACTCAGCAATGACGTTGA	
HemC22	(CA)26	F: TTTGGGTCCGTCTCATTCTC	Miyake & Yahara (2006)
		R: CCCTGGATATAATGCTTTTGTCT	
HemC25	(AC)15	F: TCTATGAGCGTGAGCGTGAG	Miyake & Yahara (2006)
		R: CGAGAGGGACACTTTTGGAA	
Yha2L04E15	(AG)17	F: CTATTGTTCCCACTAGCTGA	This study
		R: CCGAAGATGAAGACGAAGA	
Yha2L05M11	(TC)10	F: CCCATCTCTCCTTTCGAAGTC	This study
		R: GGCCATGTTTGTTATTGAGTT	
Yha3M14G09	(GA)14	F: TTCCGCAACAATAACAACCAT	This study
		R: CAGACCCGAGAACGAGCATC	
Yha3M07F01	(CT)14	F: TTCAACTCCTCATACCAACC	This study
		R: CCCAAGCCAGTGATAACA	
Yha3M10C05	(TC)7..17..(TC)9	F: AGGCATTTCACACTCTATCGT	This study
		R: CATTCGCATCTCCGTC	
Yha3M15E09	(CT)15	F: GTCGGAATATCAAACCCTAAT	This study
		R: CGAAGATGTTGCGATCCTC	

Reference

Miyake T, Yahara T (2006) Isolation of polymorphic microsatellite loci in *Hemerocallis fulva* and *Hemerocallis citrina* (Hemerocallidaceae). *Molecular Ecology Notes* **6**, 909-911.

Table S4 Definitions of prior distribution of parameters

Parameters		Distribution	Mean	SD
β_1	Tepal color	Gaussian	0	1.0E+02
γ_1		Gaussian	0	1.0E+02
β2	Scent intensity	Gaussian	0	1.0E+02
γ_2		Gaussian	0	1.0E+02
β_3	Corolla direction	Gaussian	0	1.0E+02
γ_3		Gaussian	0	1.0E+02
β_4	Stem height	Gaussian	0	1.0E+02
γ_4		Gaussian	0	1.0E+02
β_5	ASD	Gaussian	0	1.0E+02
γ_5		Gaussian	0	1.0E+02
β_6	number of visits per flower per trip bout	Gaussian	0	1.0E+02
α	Intercept	Gaussian	0	1.0E+02
r[i]	Trip bout (random effect)	Gaussian	0	τr
rp[i]	Genet ID of experimental flowers (random effect)	Gaussian	0	τrp

Table S5 Floral traits of flowers visited by swallowtail butterflies or hawkmoths

Floral traits	Visited by swallowtail butterflies			Visited by hawkmoths		
	Mean (SE)	Min.	Max.	Mean (SE)	Min.	Max.
Tepal color	22.0 (0.35)	7	23	21.4 (0.44)	3	23
Floral scent	2.29 (0.22)	0.1	6.8	3.44 (0.55)	0.1	32.9
Corolla Direction (°)	66.2 (1.63)	30	89	70.6 (1.20)	30	89
Stem height (cm)	100.0 (1.47)	74	130	96.6 (1.61)	60	127
ASD (mm)	25.1 (1.38)	0	52.2	22.2 (0.97)	2.3	44.3

Table S6 Means and 95% confidence intervals of the posterior distribution of parameters in analyses of combined fitness index calculating with Ave. X/Ave. Y = 1.

Traits	β				γ			
	Mean	SD	2.5%	97.5%	Mean	SD	2.5%	97.5%
**Swallowtail butterflies**								
Tepal color	1.934	1.771	-1.368	5.723	-3.586	3.128	-11.263	0.844
**Scent intensity**	**-17.233**	**11.490**	**-41.541**	**-0.447**	**-25.116**	**13.699**	**-53.683**	**-4.738**
**Corolla direction**	-0.034	0.333	-0.671	0.631	**-1.263**	**0.361**	**-2.009**	**-0.589**
Stem height	0.188	0.297	-0.401	0.757	-0.006	0.256	-0.526	0.490
**ASD**	**-0.777**	**0.215**	**-1.207**	**-0.367**	**0.349**	**0.141**	**0.063**	**0.626**
**Hawkmoths**								
Tepal color	1.314	0.838	-0.206	3.086	0.163	0.560	-1.001	1.246
Scent intensity	-0.140	0.804	-1.748	1.451	-0.182	0.358	-0.977	0.479
Corolla direction	-0.285	0.226	-0.733	0.166	-0.266	0.182	-0.639	0.049
Stem height	0.028	0.271	-0.502	0.561	-0.249	0.198	-0.649	0.119
ASD	-0.229	0.207	-0.624	0.188	0.024	0.153	-0.280	0.309

Bold values are the effects of the parameters classified into groups of [negative] or [positive].
